# From Scientific Curiosity to Public Enemy Number One in Six Short Months

**DOI:** 10.1371/journal.ppat.1005371

**Published:** 2016-04-14

**Authors:** Neil A. Mabbott

**Affiliations:** The Roslin Institute and Royal (Dick) School of Veterinary Sciences, University of Edinburgh, Edinburgh, United Kingdom; University of Florida, UNITED STATES

When I started my postdoctoral career in September 1995, fresh off the completion of my PhD thesis on the effects of African trypanosomes on the host immune system, I was relishing the opportunity to move to a new city, study a new pathogen, and begin my career as an immunologist. However, I was immediately surprised that my scientific friends and contemporaries, as well as members of the public, didn’t seem to share this enthusiasm. When I mentioned I was studying prions, or transmissible spongiform encephalopathies (TSEs), most people would simply respond, “Oh,” and continue whatever it was they were doing before they attempted to make small talk with a scientist. Before you do the same, I should quickly mention that prions are a group of pathogens that cause substantial damage to the brains of infected animals and humans. These diseases are currently untreatable, and they are unique in that they are caused by the simplest pathogens known to science. Prions are devoid of DNA, and they were proposed, by the Nobel Laureate Professor Stanley Prusiner, to be made up of just one molecule, the host-produced prion protein, which can mis-fold into the deadly, infectious prion particles.

I remember attending a prize lecture given by Prof. Prusiner, in which he mentioned how luck can have a big role in bringing your work to prominence. Of course, to do so, one also has to be able to recognise the opportunities, however they may arise, and have the insight to run with them. I mention this because, within six months of starting my postdoctoral career, the United Kingdom Secretary for Health made a formal statement in the Houses of Parliament about the likely link between “mad cow disease” in cattle (bovine spongiform encephalopathy; BSE) and a new human prion disease (variant Creutzfeldt-Jakob disease; vCJD). The assumption was that consumption of BSE-contaminated food may have been the source of this new prion disease in humans. All the more concerning was that this new disease was predominantly observed in young people. Ever since that announcement, it was striking to note that now, when members of the public found out what I did for a living, they didn’t walk away! Everyone had heard about prions and had their own opinions about where they came from, whether we should eat beef, and what I, as a scientist, should be doing about it all. Importantly, I had the impression that the public understood why more research was critical.

This alarming recognition that a prion disease could transmit from animals to humans brought with it many opportunities to pursue basic research into prions and prion diseases. During the intervening years, my research has been trying to answer the same simple question: How do prions spread from the gut to the brain? Addressing this issue is important because we may be able to use this information to design therapies to block the spread of prions to the brain and, in doing so, block prion disease and prevent the irreversible nerve damage it causes. Work from my own laboratory and many others around the world has made substantial progress in improving our understanding of this aspect of the disease process. Despite this, a reliable and effective therapy still eludes us. Approximately 229 cases of vCJD have been diagnosed worldwide, with the majority of these occurring in the UK. When considered in the context of other important human diseases, such as cancer and Alzheimer’s disease, the incidence of vCJD is fortunately very rare. One may therefore question why it was—and why it continues to be, in my opinion—necessary to invest in prion disease research. However, without the advances made in basic science over the years, this incidence could have been much higher. For example, data showing that prions appear to hijack cells in the body’s own immune system in order to establish disease led to the suggestion that white blood cells may harbour prions in an infected individual and might spread the disease to others via blood transfusions. Indeed, a small number of vCJD cases in the UK have since been linked to accidental transmission via infected whole blood. Based on the scientific evidence, the removal of white blood cells (leukodepletion) from blood for transfusion was introduced in the UK to reduce the possibility of accidental blood-borne prion transmission. This intervention may also have helped reduce the accidental spread of other blood-borne infectious diseases, such as viruses. Prions are also incredibly resistant to standard decontamination methods, and their potential to persist on the surfaces of surgical instruments used on infected appendixes, tonsils, etc. was highlighted. Measures have since been put in place to avoid the accidental transmission of prions via used surgical instruments.

Not all basic research discoveries will inform policy makers as to how best to reduce the incidence of prion diseases in populations, or will translate into new therapies or diagnostic tests. However, prion disease research has often led to unexpected benefits elsewhere. Early on in my career, it was evident that the prions were exploiting a rare, under-studied, population of cells in the immune system termed follicular dendritic cells. While this in itself was particularly frustrating, since little was known of their biology and few tools were available to study them, this also provided an excellent opportunity to learn more. We now understand much more about the biology of this rare population of cells—not only as they relate to prion disease but also their importance to immune function and the impact aging has on them.

While the relatively rare incidence of prion diseases may be a blessing, this does mean that, should a promising therapy or anti-prion vaccine be discovered, the economic returns are unlikely to be huge. Are other animal prion diseases (such as chronic wasting disease in deer and elk in North America, or novel strains of BSE and sheep scrapie) also able to transmit to humans? An important challenge will therefore be to engage with the pharmaceutical industry to encourage them to help to quickly turn our basic science advances into cheap, safe, and reliable anti-prion therapies for use in humans and domestic animals.

**Image 1:**
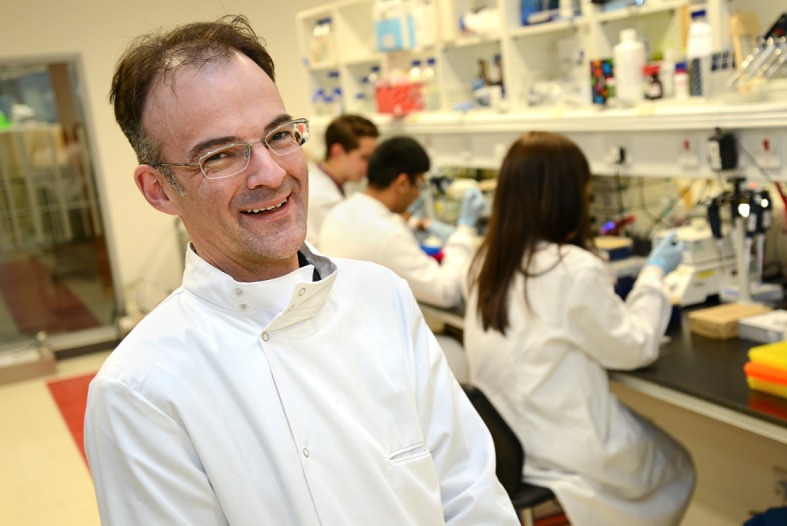
Neil A. Mabbott.

